# Unique Biomarkers of Collagen Type III Remodeling Reflect Different Information Regarding Pathological Kidney Tissue Alterations in Patients with IgA Nephropathy

**DOI:** 10.3390/biom13071093

**Published:** 2023-07-08

**Authors:** Nadja Sparding, Michaela Neprasova, Dita Maixnerova, Federica Genovese, Morten Asser Karsdal, Marek Kollar, Helena Koprivova, Zdenka Hruskova, Vladimir Tesar

**Affiliations:** 1 Nordic Bioscience, 2730 Herlev, Denmark; 2 Department of Nephrology, First Faculty of Medicine and General University Hospital, Charles University in Prague, 128 08 Prague, Czech Republic; 3 Department of Clinical and Transplant Pathology, Institute of Clinical and Experimental Medicine, 140 21 Prague, Czech Republic; 4 Institute of Medical Biochemistry and Laboratory Diagnostics, First Faculty of Medicine and General University Hospital, Charles University, 128 08 Prague, Czech Republic

**Keywords:** biomarker, fibrosis, extracellular matrix, collagen, chronic kidney disease, IgA nephropathy

## Abstract

Kidney fibrosis is the hallmark of chronic kidney disease (CKD) and is characterized by an imbalanced extracellular matrix (ECM) remodeling. Collagen type III is one of the main ECM components of the interstitial matrix of the kidney. We hypothesized that measuring three biomarkers of collagen type III reflecting different aspects of this protein turnover (C3M, C3C, and PRO-C3) may provide different information about the fibrotic burden in patients with IgA nephropathy (IgAN). We examined a cohort of 134 patients with IgAN. The three collagen type III biomarkers were measured in serum (S) and in urine (U) samples taken on the same day before kidney biopsy was performed. Biopsies were evaluated for interstitial fibrosis and tubular atrophy, according to the Banff and MEST-C scores. S-PRO-C3 and S-C3C correlated with the degree of fibrosis in the biopsy, whereas U-C3M/Cr had an inverse correlation with fibrosis. U-C3M/Cr had the highest discrimination ability for advanced fibrosis, which was maintained after adjustment for the other collagen type III biomarkers, proteinuria, and serum creatinine. The data presented in this study indicate that measuring the different fragments of the same ECM protein and in different matrices provides a variety of information regarding pathological kidney tissue alterations in patients with IgAN.

## 1. Introduction

IgA nephropathy (IgAN) is the most common primary glomerulonephritis [1,2] and occurs as a result of the deposition of immune complexes in the mesangium, causing glomerular damage [3]. The injury to the glomeruli leads to fibrosis, a significant risk factor for progression to end-stage kidney disease, which occurs in 40% of patients [4]. Kidney fibrosis is characterized by an imbalanced turnover of extracellular matrix (ECM) components such as collagens. The fibrillar type III collagen is a homotrimeric protein consisting of three alpha-1 chains and is located in the interstitium of different organs, including the kidneys [5].

The two ECM biomarker assays C3M and C3C measure the degradation of collagen type III by targeting neo-epitope fragments generated by specific protease cleavage (MMP-9 for C3M and cathepsin -B, -L, and -S for C3C). The PRO-C3 biomarker assay measures collagen type III formation by targeting the pro-peptide secreted during protein maturation. The concept of ECM degradation and formation as well as the pathological value of measuring ECM protein turnover are reviewed elsewhere [6,7]. C3M and PRO-C3 have previously been associated with disease progression and adverse outcomes in chronic kidney disease (CKD) [8,9,10], whereas C3C has been associated with disease severity in the chronic obstructive pulmonary disease [11].

Other collagen biomarkers, including the collagen type VI assay PRO-C6 (measuring collagen type VI formation and levels of endotrophin), have previously been associated with the degree of histological fibrosis in kidney biopsies from the same group of IgAN patients [12]. Recently, it was observed that the ECM biomarkers C3M, PRO-C3, PRO-C6, and LG1M (measuring the degradation of the basement membrane protein laminin) improved the prediction of kidney outcomes compared to estimated glomerular filtration rate (eGFR) and proteinuria (PU) in patients with IgAN [13]. Even though the prognostic ability of some of the collagen type III biomarkers have already been investigated, this is the first time that the association of serum and urinary levels of C3M, C3C, and PRO-C3, and the degree of histological fibrosis from patients with IgAN have been investigated.

## 2. Materials and Methods

### 2.1. Study Subjects

A total of 134 patients with biopsy-proven primary IgAN diagnosed at Charles University and General University Hospital in Prague, Czech Republic, were included in this study, and 10 healthy volunteers were enrolled as controls. All consecutive patients with IgAN diagnosed between 2011–2015, with a representative biopsy sample, available specimens in local biobank and who were willing to participate in this research study were included.

For the IgAN patients, blood and urine samples were taken on the same day before kidney biopsy was performed. Blood and urine samples from the healthy controls were collected in the same hospital following the same procedures. Informed consent was obtained from all participants and the study was conducted in compliance with the Declaration of Helsinki principles.

### 2.2. Data Collection

Clinical and laboratory data were collected or measured in the laboratories of the General University Hospital. The data included sex, age, serum creatinine (sCr), proteinuria (PU), and C-reactive protein (CRP) (all routinely measured) as well as whether the IgAN patients had diabetes mellitus or/and hypertension. The eGFR levels were calculated using the chronic kidney disease epidemiology collaboration equation [14], and the IgAN patients were stratified based on eGFR, according to the CKD classification.

### 2.3. Kidney Biopsy and Fibrosis Evaluation

Ultrasound-guided kidney biopsies were collected, evaluated for the percentage level of fibrosis (continuous variable), and classified according to the MEST-C and Banff classification for interstitial fibrosis and tubular atrophy (as previously described [12]). The Banff criteria were defined as interstitial fibrosis in ci0: ≤5%; ci1: 6–25% (mild); ci2: 26–50% (moderate); and ci3: >50% (severe) of cortical area [15]. Quantitative criteria for interstitial fibrosis and tubular atrophy according to the T-score, were defined as T0: ≤25%; T1: 26–50%; and T2: >50% [16].

### 2.4. Assays

The collagen type III biomarkers, C3M, C3C, and PRO-C3, were measured in serum (S-C3M, S-C3C, and S-PRO-C3) and urine (U-C3M, U-C3C, and U-PRO-C3) from IgAN patients and healthy controls using competitive enzyme-linked immunosorbent assays (Nordic Bioscience, Herlev, Denmark). Monoclonal antibodies were used to target one of the sequences listed in Table 1, and the biomarkers levels were measured according to the assay procedures as previously described [11,17,18,19]. Urinary creatinine levels were measured with the QuantiChrom^TM^ Creatinine kit (BioAssay Systems) and the levels of the collagen type III biomarkers measured in urine were normalized for urine creatinine (U-C3M/Cr, U-C3C/Cr, and U-PRO-C3/Cr).

### 2.5. Statistical Analyses

Spearman’s rank correlation was used to assess the association between clinical parameters and biomarker levels. The statistical differences between groups were analyzed with the Kruskal–Wallis test. Receiver operating characteristic (ROC) curve analysis was used to evaluate the discriminatory power of biomarkers for advanced fibrosis defined as a biopsy scored as ci3 (advanced fibrosis), according to the Banff score, from patients with a biopsy scored as ci0–ci2 (low–moderate fibrosis). The comparison of C-statistics was used to evaluate the superiority of the ROC curves. Log_10_-transformed data were used for parametric statistical analysis. Univariate and multivariate (with backwards elimination) linear regression analyses were performed with log_10_-transformed data to analyze the association of biomarkers with the level of fibrosis as a quantitative measure. Statistical analyses were performed using GraphPad Prism version 8.4.3 (GraphPad Software, San Diego, CA, USA) and MedCalc version 20.121 (MedCalc Software Ltd., Ostend, Belgium). *p* < 0.05 were considered significant.

## 3. Results

### 3.1. Characteristics of the Study Cohort and Association with Collagen Type III Biomarkers

A total of 134 patients with IgAN and 10 healthy controls were included in this study. The IgAN patients and healthy controls were not significant different regarding age, but the healthy control group had a higher representation of females (we do not expect the difference in age representation to influence the results). The clinical characteristics are summarized in Table 2. In the IgAN patients, uC3M/Cr had a strong inverse correlation with sCr and a strong correlation with eGFR. All other biomarkers had a modest correlation with sCr and a modest inverse correlation with eGFR. U-C3M/Cr had a modest inverse correlation with PU. Two of the urine biomarkers had a modest correlation (U-C3M/Cr inverse and U-C3C/Cr) with age. The three serum biomarkers (S-C3M, S-C3C, and S-PRO-C3) had a modest correlation with CRP (Table 3).

**Figure 1:**
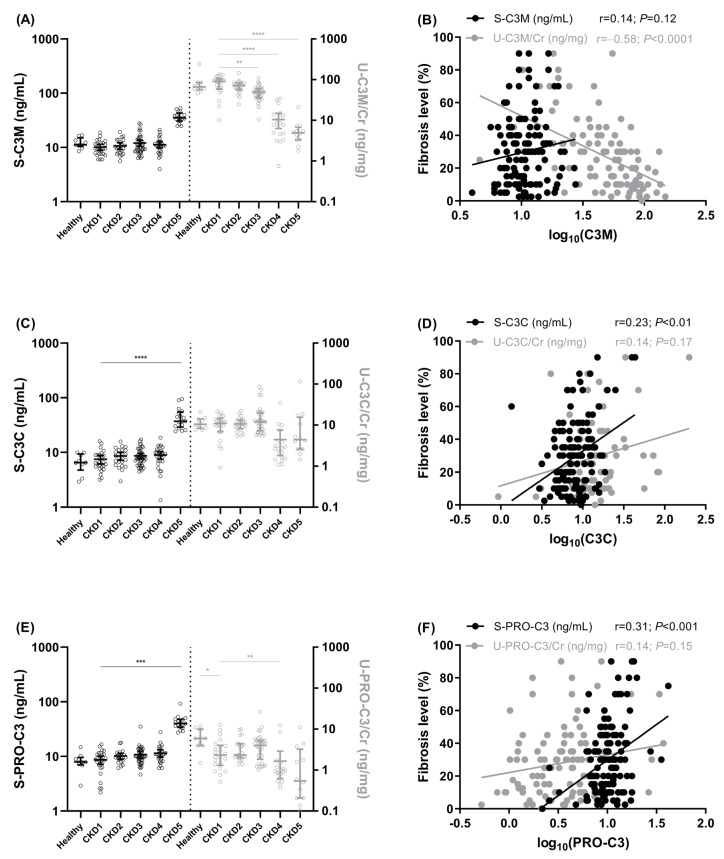
**Collagen type III biomarker levels according to CKD stages and correlation with fibrosis levels.** Serum and urinary levels of C3M (**A**), C3C (**C**), and PRO-C3 (**E**) in IgAN patients according to CKD stages (CKD1–CKD5) and in healthy controls (median with IQR and statistical differences were assessed via the Kruskal–Wallis test; * *p* < 0.05, ** *p* < 0.01, *** *p* < 0.001, **** *p* < 0.0001). Spearman’s rank correlations of serum and urinary levels of C3M (**B**), C3C (**D**), and PRO-C3 (**F**) in IgAN patients. Biomarker levels are presented on a log_10_-scale. Black symbols corresponds to black y-axis (left, ex S-PRO-C3) and grey right y-axis (ex U-PRO-C3/Cr) and the figures are divided by a dotted line in the middle.

The serum levels of the collagen type III biomarkers seemed to increase in late-stage kidney disease, even though this was only significant for S-C3C and S-PRO-C3 (Figure 1C,E). There also seemed to be a decrease in the urinary levels of the collagen type III biomarkers with increasing CKD severity, with a significant decrease in U-C3M/Cr and U-PRO-C3/Cr (Figure 1A,E).

### 3.2. Association of Collagen Type III Biomarkers with Kidney Fibrosis

The only biomarkers that significantly correlated with the level of fibrosis were U-C3M/Cr (r = −0.58, *p* < 0.0001), S-C3C (r = 0.23, *p* < 0.01), and S-PRO-C3 (r = 0.31, *p* < 0.001) (Figure 1B,D,F). U-C3M/Cr had the strongest correlation with the level of fibrosis and was the only variable with an inverse correlation (Figure 1B).

**Figure 2:**
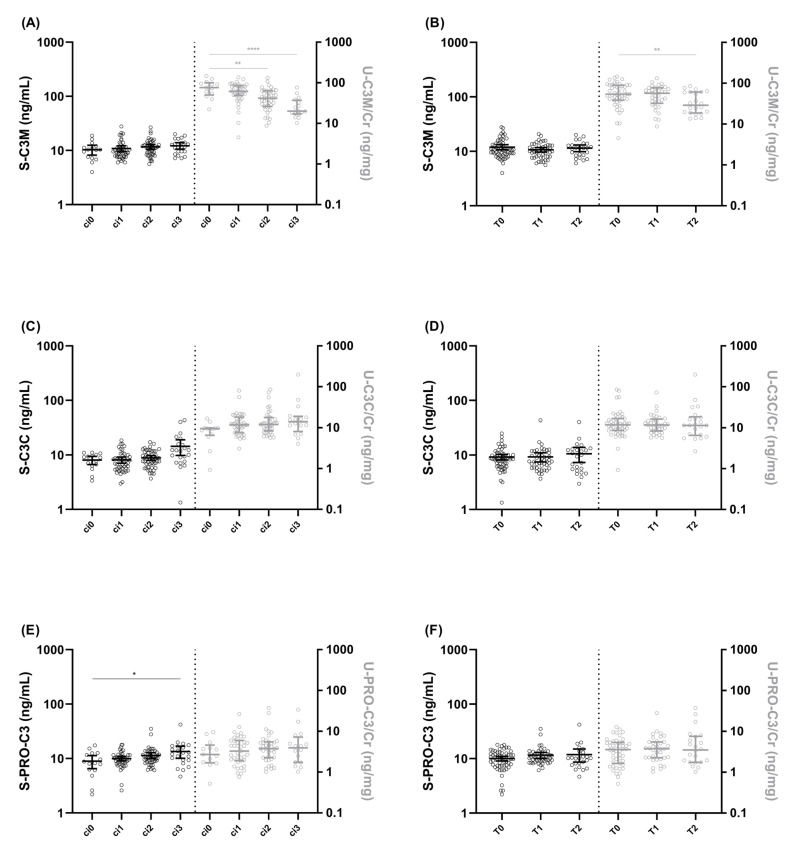
**Collagen type III biomarker levels according to interstitial fibrosis and tubular atrophy in IgAN patients.** Serum and urinary levels of C3M (**A**), C3C (**C**), and PRO-C3 (**E**) in IgAN patients, according to the Banff score (ci0–ci3). Serum and urinary levels of C3M (**B**), C3C (**D**), and PRO-C3 (**F**) according to the T-score (T0–T2). Data are presented on a log_10_-scale as median with IQR and statistical differences were assessed by Kruskal–Wallis test; * *p* < 0.05, ** *p* < 0.01, **** *p* < 0.0001. Black symbols corresponds to black y-axis (left, ex S-PRO-C3) and grey right y-axis (ex U-PRO-C3/Cr) and the figures are divided by a dotted line in the middle.

The IgAN patients were stratified into groups based on two fibrosis evaluation schemes: the Banff score and the T-score from the MEST-C classification. U-C3M/Cr levels were significantly lower in patients with biopsies scored as ci2–ci3 compared to ci0, according to the Banff system (*p* < 0.01–0.0001, Figure 2A), and in patients with biopsies classified as T2 compared to T0, according to the T-score (*p* < 0.01, Figure 2B). S-PRO-C3 levels were significantly higher in patients with biopsies classified as ci3 compared to ci0 (*p* < 0.05, Figure 2E).

The IgAN patients were divided into two groups based on the Banff score: low–moderate fibrosis (ci0–ci2) vs. advanced fibrosis (ci3). A total of 111 patients were included in the low–moderate fibrosis group and 22 patients in the advanced fibrosis group. U-C3M/Cr (AUC = 0.81, *p* < 0.0001) and S-C3C (AUC = 0.72, *p* < 0.001) were able to separate IgAN patients into advanced vs. low–moderate fibrosis groups. However, their discriminatory power was not superior to that of sCr (AUC = 0.87; *p* < 0.0001, *p* = 0.64, and *p* = 0.06 for AUC differences, respectively) (Table 4).

### 3.3. Comparison of Biomarker Association with Fibrosis Levels

The association of the collagen type III biomarkers with the level of fibrosis on a continuous scale were compared to the biomarkers of kidney function sCr and PU and hypertension and diabetes. In the univariate models, sCr, U-C3M/Cr, S-C3C, U-C3C/Cr, and S-PRO-C3 were significantly associated with the level of fibrosis, whereas PU, S-C3M, U-PRO-C3/Cr, hypertension, and diabetes were not (Table 5). U-C3M/Cr was the only variable with an inverse association with the level of fibrosis.

To investigate whether the collagen type III biomarkers added to the value of the commonly used biomarkers, the significant variables (sCr, U-C3M/Cr, S-C3C, U-C3C/Cr, and S-PRO-C3) from the univariate models in Table 5 were included in a multivariate analysis with backwards elimination. Two variables were retained in the final model: sCr (r_partial_ = 0.29, *p*<0.01) and U-C3M/Cr (r_partial_ = −0.24, *p* = 0.01) (Table 5).

## 4. Discussion

Kidney fibrosis is a hallmark of CKD and a strong predictor of progression to end-stage kidney disease [20]. To understand the processes that takes place during disease progression in CKD, it is of great value to differentiate between the process of ECM degradation and formation, rather than measuring total circulating or excreted ECM proteins. Collagen type III is a major component of the ECM and has been identified in the kidney glomerular basement membrane and the kidney interstitium [7,21]. Different biomarkers measuring specific collagen type III fragments have been developed, including the two ECM degradation biomarkers C3M [18] and C3C [11] and the ECM formation biomarker PRO-C3 [17]. Since these ECM fragments are generated by specific protease cleavage, they reflect different processes that takes place during disease progression, and they are expected to have a unique profile in different organs and diseases. The C3M biomarker is generated by MMP-9. The expression of MMP-9 has been identified in the kidneys [22], and increased MMP-9 levels were observed in patients with IgAN compared to healthy subjects [23,24]. The other degradation biomarker, C3C, is generated by cathepsin-B, -L, and -S. These proteases are also expressed in the kidneys and have been shown to regulate the kidney ECM [25]. In addition, it has been shown that cathepsin-S was upregulated in IgAN [26] and that cathepsin-L was elevated and correlated with disease severity in patients with CKD [27].

In our study, we showed that the three collagen type III biomarkers, measured in serum and in urine, had a unique association with the degree of fibrosis in kidneys as well as with other clinical parameters. All the biomarkers (S-C3M, U-C3M/Cr, S-C3C, U-C3C/Cr, S-PRO-C3, and U-PRO-C3/Cr) were associated with kidney function (sCr or eGFR), with U-C3M presenting the strongest correlation. S-C3C and S-PRO-C3 had a positive correlation and U-C3M/Cr had an inverse correlation with the level of fibrosis. S-C3C and S-PRO-C3 were also associated with inflammation (CRP) but not with PU, whereas U-C3M/Cr had an inverse association with PU but was not related to inflammation. This could indicate that the association of urinary level of C3M with kidney function may reflect the fibrotic burden in the kidneys, while the increased levels of C3C and PRO-C3 in circulation are related to the inflammatory state.

U-C3M/Cr was the ECM biomarker with the highest discrimination power for advanced fibrosis. It independently reflected the fibrotic burden after adjustment for markers of kidney functions. The fact that U-C3M/Cr and sCr were the only biomarkers retained in a final model for the fibrotic burden in biopsies from patients with IgAN may indicate that these markers are both strongly related to the fibrotic burden but describe different processes, and thus, U-C3M/Cr adds to the information provided by sCr. This will need to be evaluated in larger cohorts and in CKD patients with different etiologies.

This presented study are limited by the lack of data for the expression of collagenases and their association with fibrosis in the kidney. This study is further limited by the relatively small number of IgAN patients and the fact that the association of the biomarkers with the degree of fibrosis was only investigated in patients with IgAN. The association of the biomarkers with the degree of fibrosis, especially U-C3M/Cr, needs to be confirmed in a larger IgAN cohort and in cohorts with other etiologies of CKD.

The presented data indicate that measuring different fragments of the same ECM protein and in different matrices provide different information regarding pathological kidney tissue alterations in patients with IgAN. This highlights the importance of measuring the right epitope in different organs and diseases. Among the investigated biomarkers, U-C3M/Cr appears to be a promising non-invasive biomarker of fibrosis that could be used as a supplement to kidney biopsies.

## Figures and Tables

**Table 1:** Collagen type III biomarkers.

Name	Description	Target Gene	Target Sequence	Biological Function	References
C3M	A fragment of type III collagen released by MMP-9	COL3A1	KNGETGPQGP	Interstitial matrix degradation	[18,19]
C3C	A fragment of type III collagen released by cathepsin-B, -L, and -S	COL3A1	GLPGTGGPPG	Interstitial matrix degradation	[11]
PRO-C3	N-terminal type III collagen pro-peptide released by ADAMTS-2	COL3A1	PTGGQNYSP	Interstitial matrix formation	[17,19]

ADAMTS, a disintegrin and metalloproteinase with thrombospondin motifs; MMP, matrix metalloproteinases.

**Table 2:** Characteristics and biopsy assessment.

Variables	Healthy	IgAN	N (IgAN)
N	10	134	134
Women, %	90 ****	31	134
Age, year	41 (39–42) ^ns^	43 (32–54)	134
sCr, mg/dL	NA	1.7 (1.0–2.4)	134
eGFR, mL/min/1.73 m^2^	NA	46 (26–82)	134
PU, g/day	NA	1.7 (0.8–3.2)	131
CRP, mg/L	NA	2.5 (1.0–4.8)	126
CKD stage (1,2,3,4,5), %	NA	19, 16, 31, 20, 13	134
Level of fibrosis, %	NA	30 (10–40)	128
Banff score (ci0, ci1, ci2, ci3), %	NA	11, 34, 38, 17	133
T-score (T0, T1, T2), %	NA	45, 36, 19	130
Hypertension ^a^, %	NA	64	134
Diabetes ^b^, %	NA	8	134

Continuous variables are expressed as median (IQR) and categorial variables as %. Significant difference between the healthy and the IgAN groups was assessed with Mann–Whitney or chi-squared tests, where ^ns^ = not significant, and **** *p* < 0.0001 compared to IgAN. CKD, chronic kidney disease; CRP, C-reactive protein; eGFR, estimated glomerular filtration rate; NA, not available; PU, proteinuria; sCr, serum creatinine. ^a^ These patients had well-corrected hypertension. ^b^ These patients had well-compensated diabetes mellitus.

**Table 3:** Spearman’s rank correlation coefficients of fibrosis levels, C3M, C3C, and PRO-C3 with clinical parameters.

	Variables	Level of Fibrosis	S-C3M	U-C3M/Cr	S-C3C	U-C3C/Cr	S-PRO-C3	U-PRO-C3/Cr
IgAN	Age	0.11	0.04	−0.30 **	0.04	0.22 *	0.08	0.13
	sCr	0.64 ****	0.18 *	−0.70 ****	0.33 ***	0.20 *	0.31 ***	0.26 **
	eGFR	−0.63 ****	−0.18 *	0.66 ****	−0.34 ***	−0.24 *	−0.36 ****	−0.34 ***
	PU	0.18 *	−0.05	−0.32 **	0.10	0.06	0.05	0.07
	CRP	0.02	0.29 **	−0.08	0.34 ***	0.13	0.18 *	0.07

Statistical significance; * *p* < 0.05, ** *p* < 0.01, *** *p* < 0.001, **** *p* < 0.0001. CRP, C-reactive protein; eGFR, estimated glomerular filtration rate; PU, proteinuria; sCr, serum creatinine.

**Table 4:** Discriminatory power for advanced fibrosis.

Biomarkers	AUC	95% CI	*p*-Value	Sensitivity	Specificity	Criterion	Correct Classification
sCr	0.87	0.80–0.92	<0.0001	77.3	87.4	>2.6	85.0%
S-C3M	0.61	0.52–0.69	0.11	59.1	65.8	>11.5	64.7%
U-C3M/Cr	0.81	0.72–0.88	<0.0001 (vs. sCr *p* = 0.64)	81.3	71.4	≤38.5	72.9%
S-C3C	0.72	0.64–0.79	<0.001 (vs. sCr *p* = 0.06)	54.6	83.8	>11.7	78.2%
U-C3C/Cr	0.58	0.48–0.67	0.35	50.0	73.6	>14.6	70.1%
S-PRO-C3	0.64	0.55–0.72	0.06	54.6	76.6	>11.9	72.2%
U-PRO-C3/Cr	0.52	0.42–0.61	0.86	25.0	86.8	>7.9	77.6%

ROC criteria: Banff score ci3 (advanced fibrosis) vs. ci0–ci2 (low–moderate fibrosis). AUC, area under the curve; CI, confidence interval.

**Table 5:** Association of biomarker levels with the extent of fibrosis.

Linear Regression	Univariate	Multivariate
**Variables**	**r**	**r_partial_**
sCr	0.62 ****	0.29 **
PU	0.14	NR
S-C3M	0.13	NR
U-C3M/Cr	−0.51 ****	−0.24 **
S-C3C	0.24 **	NR
U-C3C/Cr	0.23 *	NR
S-PRO-C3	0.30 ***	NR
U-PRO-C3/Cr	0.18	NR
Hypertension, yes	0.17	NR
Diabetes, yes	−0.15	NR

Univariate and multivariate (with backwards elimination) linear regression analysis. Variables and outcome (level of fibrosis (%)) used for the regression analysis were log_10_ transformed for the linear regression analysis. Statistical significance; * *p* < 0.05, ** *p* < 0.01, *** *p* < 0.001, **** *p* < 0.0001. NR, not retained in the model; PU, proteinuria; sCr, serum creatinine.

## Data Availability

The data that support the findings of this study are available from the corresponding author upon reasonable request.
